# Comparison of the Efficacy of the Cartridge-Based Nucleic Amplification (CBNAAT)/Xpert Test and Histology of Genital Tissues in Diagnosing Female Genital Tuberculosis

**DOI:** 10.7759/cureus.15000

**Published:** 2021-05-13

**Authors:** Vaibhav Kanti, Shikha Seth, Sumedha Gupta, Vandana Verma, Amit Singh, Geeta Maurya, Adesh Kumar

**Affiliations:** 1 Obstetrics and Gynecology, Uttar Pradesh University of Medical Sciences, Etawah, IND; 2 Obstetrics and Gynecology, Government Institute of Medical Sciences​ (GIMS), Greater Noida, IND; 3 Obstetrics and Gynecology, Uttar Pradesh Rural Institute of Medical Sciences and Research, Etawah, IND; 4 Microbiology, Uttar Pradesh University of Medical Sciences, Etawah, IND; 5 Pathology, Uttar Pradesh University of Medical Sciences, Etawah, IND; 6 Pulmonary Medicine, Uttar Pradesh University of Medical Sciences, Etawah, IND

**Keywords:** female genital tuberculosis, menstrual irregularities, histopathology examination, infertility, cbnaat/xpert/rif assay

## Abstract

Background

Diagnosing female genital tuberculosis (FGTB) is very difficult by routine laboratory investigations. Collecting tissues from genital structures, especially from tubes for histology, is impossible. The cartridge-based nucleic amplification (CBNAAT)/Xpert RIF test is a new polymerase chain reaction (PCR)-based method that is quick and may diagnose FGTB from any tissue type; however, it should not be contaminated with blood. This study was conducted to compare the efficacy of CBNAAT and the histology of genital tissue in suspected cases.

Materials and methods

This was a prospective study of the diagnostic efficacy of 91 cases of suspected FGTB randomly selected from March 2018 to September 2019 at a rural tertiary care center. Endometrial tissue collected in 86 patients (59 infertility, 27 menstrual irregularities) and tubal/peritoneal tissue from hysterectomy or laparotomy specimens of five participants who underwent surgery were sent for histopathological analysis and CBNAAT and the results were evaluated and compared.

Results

There were 59 (64.83%) and 32 (35.2%) cases of infertility and menstrual irregularities, respectively. Primary infertility (38; 41.75%) was the most common complaint. Endometrial biopsies (EB) of two (2.23%) cases were found positive for tuberculosis (TB) both on histopathological examination (HPE) and CBNAAT. In addition, both patients had primary infertility. Of the 32 cases with menstrual abnormalities (27 EB and three tubal tissue, two peritoneal and nodular tissue), none were found to be positive for TB on HPE or CBNAAT. A highly significant association was found between histopathology and CBNAAT (p<0.0001) in the endometrial tissue of infertile patients. Sensitivity, specificity, positive predictive value (PPV), and negative predictive value (NPV) were 100% for CBNAAT, with reference to histopathology.

Conclusion

We recommend CBNAAT for the early detection of FGTB, with the added advantage of early results, minimal technical expertise, and detection of drug-resistant tuberculosis (TB).

## Introduction

Female genital tuberculosis (FGTB) leads to various non-specific symptoms in women, ranging from infertility to menstrual irregularities and pelvic pain [1]. The incidence of FGTB ranges from 1%-19% in different parts of India [2]. Infertility is the most common symptom of FGTB, and the incidence of FGTB in infertility varies from 3%-16% in India [3]. Given the high prevalence of diseases in patients with infertility and menstrual disorders, a high degree of clinical suspicion for FGTB should be considered. Therefore, detailed history, systemic examination, and series of investigations are needed [3]. Many diagnostic modalities have been developed although diagnosing FGTB remains challenging due to its paucibacillary nature. Laboratory investigations, such as serology and microscopy, have little value in diagnosis, and culture takes a long time. Polymerase chain reaction (PCR) has high sensitivity but low specificity [4]. Histopathology of genital/endometrial tissue may detect tuberculosis (TB) by granulomatous lesions; however, it is not diagnostic because granulomas may also be found in other infectious diseases [5]. Cartridge-based nucleic amplification test (CBNAAT) is a recently introduced polymerase chain reaction (PCR)-based method for detecting TB, which can provide results within 100 min. Several studies have utilized the new PCR-based Xpert MTB (*Mycobacterium tuberculosis*)/CBNAAT for the diagnosis of other types of extrapulmonary TB. Only a few studies have utilized CBNAAT in the diagnosis of FGTB [6]. CBNAAT can be used in low resource settings to facilitate patients’ access to early and accurate diagnoses. Therefore, the present study was conducted to establish a correlation between the histopathology of various genital tissues with CBNAAT in the diagnosis of FGTB.

## Materials and methods

The study was conducted in the department of obstetrics and gynecology, Uttar Pradesh University of Medical Sciences (UPUMS), Saifai, Etawah, in collaboration with the department of pathology and microbiology from March 2018 to September 2019 (one year six months), after obtaining ethical clearance. After a detailed history, thorough clinical examination, and investigations, such as complete blood count, chest radiography, human immunodeficiency virus (HIV) I and II, and abdominal and pelvic sonogram, clinically suspicious patients of FGTB were randomly selected and included in the study after obtaining informed consent. All data relating to the demographic profile of symptomatic cases, specific clinical symptoms, and signs based on clinical findings, imaging, laparotomy, or laparoscopy were also noted to establish the prevalence of different signs and symptoms of FGTB. Women presenting with primary or secondary infertility or blocked tubes on hysterosalpingography (HSG) and those who complained of menstrual disorders, such as secondary amenorrhea, oligomenorrhea, and menorrhagia with idiopathic causes, were included. Patients with a history of the complete course of anti-tuberculosis treatment intake in the past or with intrauterine fibroids, polyps, or endometriosis on ultrasonography (USG) were excluded from the study. Patients undergoing HPE and CBNAAT investigations were randomly selected based on clinical suspicion of TB. Specimens from suspected patients were collected in the form of premenstrual endometrial biopsy (EB) in cases of infertility and menstrual disorders. During operative procedures, such as hysterectomy or laparotomy/laparoscopy, if any tubercular lesions were suspected, specimens in the form of tubal tissue, peritoneal tissue, or any suspicious nodules were obtained. Tissue collected from each participant was divided into two containers: one for histopathological analysis in a 10% formalin vial and one for CBNAAT in sterile containers with normal saline. Endometrial biopsy was performed in 59 infertility and 27 menstrual disorder cases. Tubal tissue was sent in three cases with menorrhagia undergoing hysterectomy, with findings of tuberculous lesions on tubes per operative. Two cases of oligomenorrhea had tubo-ovarian masses in which peritoneal tissues and suspicious nodules were sent as specimens. Comparison of the efficacy and diagnostic ability of histopathological examination (HPE) with CBNAAT (HPE was taken as the gold standard test to detect TB) in clinical suspects of FGTB was performed using appropriate statistical analysis.

## Results

A total of 91 cases were enrolled in this study over 18 months. Most participants were 21-30 years old. Specimens were collected from the endometrium in 59 (64.83%) infertility cases and from 27 (29.6%) with menstrual irregularities. In five cases, tissue other than the endometrium (5.5%) was sent. In two of five cases with tubo-ovarian masses on laparotomy, peritoneal and suspicious nodular tissue were obtained, and in the remaining three cases, tubal tissue with tubercles was obtained during hysterectomy. All the specimens obtained were sent for both HPE and CBNAAT.

The mean age of participants with infertility was 26.7 years while that for participants with menstrual irregularities was 35.7 years. The majority of 41 (45%) cases were nullipara, and 52 (57%) cases had lower socioeconomic status (Table 1). All cases were HIV negative. No patient had any comorbid conditions. The maximum number of infertility cases enrolled was primary infertility (41.75%). Secondary infertility was observed in 23% of the cases (Table 2). The most common menstrual irregularity was menorrhagia in 23 (25.3%) cases, followed by oligomenorrhea in six (6.6%) cases. Amenorrhea was found in three (3.3%) cases. A history of TB was found in six (6.6%) patients while family history was found in five (5.5%) of the total cases (Table 1).

**Table 1:** Distribution of patients according to demographic profile TB: tuberculosis

Demographic characters		Number of patients (n=91)	Percentage	Mean ± SD
Age group (in years)	21-25	34	37.4	
	26-30	29	31.9	
	31-35	11	12.0	29.8 years
	36-40	5	5.5	
	41-50	12	13.2	
Parity	Nullipara	41	45.0	
	Primipara	25	27.5	1.07
	Multipara	25	27.5	
History of TB	Past history	6	6.6	
	Family history	5	5.5	
	No history	80	88.0	

**Table 2:** Distribution of patients according to presenting symptoms

Presenting symptoms	Number of patients (n=91)	Percentage
Amenorrhea	3	3.3
Menorrhagia	23	25.3
Oligomenorrhea	6	6.6
Primary infertility	38	41.7
Secondary infertility	21	23.0

The investigations and procedures performed on the participants, along with their findings, are shown in Table 3. Chest radiography was normal, with no significant findings in all cases, and USG findings were normal in all except two cases with menstrual irregularities (oligomenorrhea) showing a unilateral complex tubo-ovarian mass. These two patients underwent laparotomy (unilateral adnexectomy), and peritoneal tissues with nodular lesions were sent for both tests. HSG was performed in 34 of 59 patients enrolled for infertility. Bilateral spillage of dye was seen in 76.5% of cases, and these cases had no findings suggestive of FGTB. Five cases showed one side, and three had a bilateral tubal block on HSG. One patient with a right-sided tubal block was later found to be positive for both HPE and CBNAAT. Another patient had a normal HSG but was positive for FGTB on both HPE and CBNAAT.

**Table 3:** Distribution of patients according to the results of the investigation

Investigations	Reports	Number of patients (n=91)	Percentage
Chest radiograph PA view	Normal	91	100%
Ultrasonography	Tubo-ovarian mass	2	2.1
	Normal	89	97.9
Hysterosalpingography (34)	Normal	26	82.3
	Tubal block	8	6.6
CBNAAT for TB	Positive	2	2.1
	Negative	89	97.9

Twenty of 59 infertility cases underwent laparoscopy, and five of 91 cases underwent laparotomy. Only two of 20 cases of infertility with cornual block were found to be suspicious of TB on laparoscopy. Still, the endometrial sample HPE showed non-specific endometritis, secretory endometrium, and chronic endometritis, and CBNAAT reports were negative for TB.

As shown in Table 4, endometrial hyperplasia was found in three (3.5%) cases, non-specific endometritis in two (2.3%), proliferative epithelium in 48 (55.8%), and secretory epithelium in 31 (36%) cases. There were two cases of tuberculous endometritis, which was 2.32% of the total endometrial biopsies, 3.4% of the total infertility participants, and 2.2% of the total clinical suspects. These cases were also positive for CBNAAT. Three samples of tubal tissue with tubercles and, in two cases, peritoneal tissues with suspicious nodules were obtained and sent for histopathological examination from cases that underwent hysterectomy/laparotomy. All five were found to be negative for TB on histopathology and CBNAAT.

**Table 4:** Spectrum of histopathological findings

Histopathology findings of the endometrium	Total number of patients in which endometrium was taken (n=86)	Percentage
Endometrial hyperplasia	3	3.3%
Nonspecific endometritis	2	2.2
Proliferative epithelium	45	52.7
Secretory epithelium	34	39.6
Tubercular endometritis	2	2.2

As the cases that showed findings suggestive of tuberculosis in histopathology (two cases) also showed positive results for TB in the CBNAAT/Xpert RIF assay; the sensitivity, specificity, positive predictive value (PPV), negative value (NPV), and diagnostic accuracy were 100% for CBNAAT regarding histopathology and the measure of agreement (kappa) was 1.00. A highly significant association was found between the histopathology of endometrial tissue and CBNAAT (p<0.0001). However, no association was found between the histopathology of tubal or peritoneal tissue and the corresponding CBNAAT (Table 5).

**Table 5:** Association of histopathological findings with CBNAAT for tuberculosis CBNAAT: cartridge-based nucleic amplification test

Histopathology (for tuberculosis)	CBNAAT results for tuberculosis (n=91)		Total number of patients
	Negative	Positive	
Negative (n=89)	89	0	89
Positive (n=02)	0	2	2
Total	89	2	91

As the cases that have shown tubercular finding in histopathology (two cases) also showed positive results for tuberculosis in CBNAAT/Xpert RIF assay; sensitivity, specificity, PPV, NPV, and diagnostic accuracy were 100% for CBNAAT with reference to histopathology and the measure of agreement (kappa) was 1.00. A highly significant association was found between the histopathology of endometrial tissue and their CBNAAT (p<0.0001), while association could not be found between the histopathology of tubal or peritoneal tissue and their CBNAAT (Table 5).

## Discussion

Collecting tissue is not always possible from genital structures, especially from tubes for HPE and CBNAAT in young women suspected of disease, especially when they present with infertility. Although laparoscopy is a routine protocol in infertility cases, it is an invasive procedure and requires hospitalization, anesthesia, and a good endoscopic OT setup for diagnostic purposes. The CBNAAT/Xpert RIF assay is a new PCR-based method that is quick and may diagnose FGTB from any kind of genital tissue, but it should not be contaminated with blood. There is a need to find other less invasive, simple methods to collect samples from the pelvis or tubes of patients suspected of FGTB in our country, where the prevalence of TB is very high. We conducted a study to compare the efficacy of CBNAAT with the histology of genital tissue in suspected cases. Several studies have utilized the new PCR-based Xpert MTB/CBNAAT for diagnosing other types of extrapulmonary TB. However, only a few studies have utilized CBNAAT in the diagnosis of FGTB, and, therefore, very little data on the role of CBNAAT in FGTB are available.

Ninety-one clinical suspects of FGTB, 59 (64.83%) of infertility, and 32 (32.2%) of menstrual irregularities were recruited in our study. The mean age of participants was 26.7 years in infertility and 35.7 years in the group with menstrual irregularity. The age and clinical presentations were consistent with those of other similar studies [3,7]. Specimens were collected from all patients suspected of FGTB based on their symptomatology and preoperative findings. Endometrial tissue in 86 cases (59 infertility, 27 menstrual irregularities) and tubal/peritoneal tissue from hysterectomy/laparotomy specimens of patients who underwent surgery in five cases were collected and sent for histopathological analysis and CBNAAT. A history of TB was found in six (6.6%) cases while family history was found in five (5.5%) cases. No cases positive for FGTB by HPE and CBNAAT had close contact with TB patients in the family. In the study conducted by Thangappah, 5.5% of patients had a history of TB, similar to our study [7].

Our study found an incidence of FGTB of 3.4% in primary infertility cases by both HPE and CBNAAT of endometrial tissue. Even a normal case in history, examination, and imaging was found to have tuberculous endometritis. All patients positive for CBNAAT were rifampicin-sensitive, which excluded MDR-TB. In both positive cases for TB in CBNAAT, very low MTB was detected. Most previous studies detected TB on endometrial slides, but only a few have diagnosed FGTB on CBNAAT/Xpert [2-4,8]. Thangappah found that 6.9% of the endometrial samples were positive [7]. Goel et.al. found that 2.6% of patients had histopathological slides of the endometrium suggestive of TB [8]. The CBNAAT score in the study conducted by R. Saxena was 1.6%, which was less than that in our study [9]. Farhan et al. reported an improvement in the detection rate of TB using the CBNAAT/Xpert method and found 8.05% positive cases [3]. A study by Roopakshi et al. in 81 infertility cases showed 1.23% positivity for tubercular endometritis, but Xpert/CBNAAT scored negative in their study [4].

Chest radiography findings were within normal limits in all cases (100%). HSG was performed in 34 cases of 59 infertility cases. Of the 34 HSG, eight showed tubal block and one tubal block case was diagnosed as having tubercular endometritis by both HPE and CBNAAT. As we could not collect the sample tissue or material from the tubes in all cases, we cannot be sure about the absence of tubercular disease in such cases.

USG findings were normal in 89 (97.80%) patients in our study. Two patients had unilateral tubo-ovarian masses. One mass had a size of approximately 5 × 7 × 6 cm and another 4 × 3 × 2 cm. In contrast to our study, Thangappah et al. found evidence of calcification by ultrasound or X-ray abdomen was seen in five (6.9%) women and cornual blocks were seen in 35 (54.2%) cases on hysterosalpingogram [7].

Exploratory laparotomy was performed in two cases with a tubo-ovarian mass, and peritoneal tissue with nodules was obtained in both cases. Three suspects of FGTB were found on routine hysterectomy by white or caseous tubercle-like lesions on tubes and para-tubal areas, where tubal tissues were collected. All were negative for TB on CBNAAT while histopathological examination of tubal tissue specimens showed epithelial hyperplasia, and peritoneal tissues showed inflammatory smears with histiocytes and foci of hyalinization and calcification.

On histopathological examination, two (3.3%) cases in our study were diagnosed as having tuberculous endometritis and were also found to be positive for TB in CBNAAT. Garg et al. found one case positive for tubercular endometritis in 81 patients with primary infertility [4]. In our study, both cases positive for HPE and CBNAAT for FGTB presented with complaints of primary infertility. Our study found two cases of tuberculous endometritis on histopathology, which were also positive on CBNAAT. All other patients were negative for TB, both on histopathology and CBNAAT. Therefore, a highly significant association was found between histopathology and CBNAAT (p<0.0001).

The measure of agreement (kappa) was 1.00 for CBNAAT, with reference to histopathology, while sensitivity, specificity, PPV, NPV, and diagnostic accuracy were 100% for CBNAAT with respect to histopathology. Very few studies have been conducted on CBNAAT/GeneXpert as a diagnostic modality for endometrial TB; hence, not much data could be made available to compare our results.

This study is 'one of a kind' with a prospective design; however, it has a few limitations such as small sample size (91 patients). Thus more retrospective and prospective studies with a larger sample size are required to support the study.

## Conclusions

India is a highly prevalent zone for TB; therefore, it is highly suspected when considering the diagnosis. To date, none of the available tests can detect all cases of genital TB. The CBNAAT/Xpert MTB/RIF assay can be used for the early detection of FGTB and provides the added advantage of early results, minimal technical expertise, and the detection of drug-resistant TB. This is because it has a higher detection rate as compared to conventional methods. Moreover, larger multicenter studies are required to suggest the most appropriate and cost-effective test for the diagnosis of genital TB.
